# Graviola (*Annona muricata*) attenuates behavioural alterations and testicular oxidative stress induced by streptozotocin in diabetic rats

**DOI:** 10.1371/journal.pone.0222410

**Published:** 2019-09-11

**Authors:** Abdel-wahab A. Alsenosy, Ali H. El-Far, Kadry M. Sadek, Safinaz A. Ibrahim, Mustafa S. Atta, Ahmed Sayed-Ahmed, Soad K. Al Jaouni, Shaker A. Mousa

**Affiliations:** 1 Department of Biochemistry, Faculty of Veterinary Medicine, Damanhour University, Damanhour, Egypt; 2 Department of Animal Husbandry and Animal Wealth Development, Faculty of Veterinary Medicine, Damanhour University, Damanhour, Egypt; 3 Department of Physiology, Faculty of Veterinary Medicine, Kafrelsheikh University, Kafrelsheikh, Egypt; 4 Department of Anatomy and Embryology, Faculty of Veterinary Medicine, Menoufia University, Menoufia, Egypt; 5 Hematology/Pediatric Oncology, King Abdulaziz University Hospital and Scientific Chair of Yousef Abdullatif Jameel of Prophetic Medicine Application, Faculty of Medicine, King Abdulaziz University, Jeddah, Saudi Arabia; 6 Pharmaceutical Research Institute, Albany College of Pharmacy and Health Sciences, Rensselaer, NY, United States of America; National Institutes of Health, UNITED STATES

## Abstract

Oxidative stresses intensify the progression of diabetes-related behavioural changes and testicular injuries. Graviola (*Annona muricata*), a small tree of the *Annonaceae* family, has been investigated for its protective effects against diabetic complications, oxidative stress, and neuropathies. This study was planned to investigate the effects of graviola on behavioural alterations and testicular oxidative status of streptozotocin (STZ; 65 mg/kg)-induced diabetic rats. Forty adult male Wistar rats were equally allocated into four groups: control (received normal saline 8 ml/kg orally once daily), diabetic (received normal saline orally once daily), graviola (GR; received 100 mg/kg/day; orally once daily), and diabetic with graviola (Diabetic+GR; received 100 mg/kg/day; once daily). Behavioural functions were assessed using standard behavioural paradigms. Also, oxidative statuses of testis were evaluated. Results of behavioural observations showed that diabetes induced depression-like behaviours, reduction of exploratory and locomotor activities, decreased memory performance, and increased stress-linked behaviours. These variations in diabetic rats were happened due to oxidative stress. Interestingly, treatment of diabetic rats with graviola for four weeks alleviated all behavioural changes due to diabetes. Also, rats in graviola-treated groups had greater testicular testosterone and estradiol levels compared with diabetic rats due to significant rise in testicular acetyl-CoA acetyltransferase 2 expression. In the same context, graviola enhanced the antioxidant status of testicular tissues by significantly restoring the testicular glutathione and total superoxide dismutase that fell during diabetes. In addition, Graviola significantly decreased the expression of apoptotic (*Bax*) and inflammatory (*interleukin-1β*) testicular genes. In conclusion, these data propose that both the hypoglycemic and antioxidative potential of graviola are possible mechanisms that improve behavioural alterations and protect testis in diabetic animals. Concomitantly, further clinical studies in human are required to validate the current study.

## Introduction

Diabetes mellitus (DM) is a metabolic disorder accompanied by hyperglycemia resulting from insulin production deficiency, insulin resistance, or both leading to a variety of complications [1]. DM can cause central nervous system damages, which lead to neurodegeneration and multiple alterations in the structure and biological functions of the brain [2,3]. Diabetic-related changes increase the hazard of a range of neurobehavioral turbulences such as psychosis, anxiety, depression, cognition, and locomotor disturbances [4].

Oxidative stress has a serious basis role in the progression of diabetes occurrence and its complications [5,6]. Hyperglycemia can enforce oxidative imbalance, which raises free radicals’ production and reduces the antioxidant defences that then cause serious cellular damage to cellular structures including lipids, amino acids, nucleic acids, and proteins [7]. Regarding reproduction, oxidative stress is a major factor in male infertility [8,9]. Reactive oxygen species (ROS) cause lipid peroxidation and DNA fragmentation, disrupting both the survival of lipids and DNA and their supportive role of normal embryonic development in isolated spermatozoon [10]. So, antioxidants may be useful in the treatment of infertile males [11].

Development of herbal products with antidiabetic properties and fewer side effects is of great importance to contributing to control of diabetic alterations [12]. Herbal therapies have been used traditionally in many parts of the world. Graviola (*Annona muricata* L.), which belongs to the family of *Annonaceae*, is an evergreen tree species used as traditional medicine. The plant has core pharmacological activities that include antileishmanial [13], antiplasmodial [14], injure healing [15], antioxidant [16], and anticancer [17] activities. Graviola leaves are the most valuable parts of the tree. Many researchers were identified and isolated about two hundred chemical compounds from graviola; the most important being alkaloids, phenols and acetogenins [18]. Also, it has acetogenins-containing compounds, namely bulatacin, asimisin, and squamosin [19]. Also, Alitonou *et al* [20] detected numerous phenols, flavonoids, vitamins, and carotenoids in graviola leaves. Argentinine, cinnamic acid, coumarid acid, catechin, epicatechin, genistein, quercetin, and gallic acid were isolated from graviola and they possessed antioxidant potentials in normal and immortalized human cell lines [21,22].

Graviola leaves are also believed to stabilize the blood sugar level in the normal range that is very useful for diabetes management. Numerous studies have investigated that graviola leaf has antihyperglycemic activity and revealed regeneration of pancreatic islet in stained pancreatic sections of diabetic rats [23]. The present study was conducted to evaluate the protective effects of graviola leaves on the behavioural and testicular alterations in experimentally induced diabetes in rats.

## Material and methods

### Chemicals

Streptozotocin (STZ), glucose, 0.1 M citrate buffer, phosphate buffered saline, and sodium chloride solution (0.9%) were purchased from Sigma-Aldrich (Sigma Chemical Co., St. Louis, MO, USA). Total RNA extraction and SYBR Green Master Mix kits were purchased from Qiagen Co. (Düsseldorf, Germany). cDNA kit was obtained from Promega Co., Madison, WI, USA. Graviola Dry Extract^®^ (product code: 912943735) was purchased from Origini Naturali Company (Quarrata, Pistoia, Italy).

### Ethics statement

The study was approved by the Ethics Committee of Animal Care of the Faculty of Veterinary Medicine, Damanhour University, Egypt and based on the “NIH Guide for the Care and Use of Laboratory Animals”. All precautions were followed to decrease animal suffering throughout the experiments.

### Experimental design

Forty adult male Wistar rats (140 ± 20 g) were bought from the research institute of Alexandria University, Egypt. They were maintained in standard laboratory conditions with 12 h light/dark cycle. Food pellets and tap water were accessed *ad libitum* for rats as stated Atta *et al* [24]. After one week, the rats were randomly allotted into four groups (*n* = 10 per group in three replicates each) including normal control (Control), graviola-treated (GR), Diabetic, and diabetic treated with graviola (Diabetic+GR) groups. Rats in GR and Diabetic+GR groups were supplemented with 100 mg graviola dissolved in normal saline 0.9% per kg BW daily using gavage needles orally. All treatments were continued for 4 weeks. Both control and diabetic groups were treated similarly with normal saline (0.9%) as adjuvant. The dose of graviola applied in this experiment was determined following the study of Florence *et al* [25]. All behavioural tests were conducted at the end of the 2^nd^ and 4^th^ weeks. After 4 weeks, the rats were anesthetized with intravenous injection with sodium pentobarbital (30 mg/kg) and sacrificed for proper sampling and subsequent analysis. Body weights were recorded at both the start and the end of the study.

### Diabetes induction

Experimental diabetes was brought on by intraperitoneal injection of rats with a single dose of STZ (65 mg/kg) dissolved in citrate buffer (0.1 M, pH 4.5). Seventy-two h post STZ injection, serum glucose levels were determined using a glucose oxidase colorimetric method in a tail vein blood sample (Biodiagnostic Co., Giza, Egypt). Rats that had blood glucose levels more than 250 mg/dl were considered diabetic (citrate buffer was injected in non-diabetic groups).

### Open field test

Open field test (OFT) was carried out to observe exploratory and locomotor changes [26]. OFT was basically comprised of large square chamber 56 × 56 cm. Chamber walls and floor were wood. The open field arena was divided into a grid of equally sized areas by lines drawn on the chamber floor (12 squares) for visual scoring of activity by the experimenter. The animals were individually placed in the centre of the chamber, open field session lasted for 5 min, and during this time the observer recorded the number of crossing responses, rearing behaviour, grooming activity, and defecation [27].

### Elevated plus maze

Elevated plus maze (EPM) was carried out to study the locomotor activity and anxiety-related behaviour as described by Pellow *et al* [28]. The EPM device consisted of two sets of opposing arms of wood with approximately: open arms, 30 × 5 cm; closed arms, 30 × 5 cm; surrounded by 15-cm-high walls that were elevated 40 cm above the floor. The device was placed in a separate room without objects that could give signals and disturb patterns of behaviour. Rats were located on the central platform (5 × 5 cm) of the maze, opposing the closed arm and allowed to explore the maze for 5 min. The rats’ pathways were inspected by observer to determine the behavioural parameters including frequencies of entries into open arms (EOA), frequencies of entries into closed arms (ECA), percentage of open arms entries (%EOA), frequencies of grooming, frequencies of rearing, and frequencies of defecations. The maze was disinfected with ethanol (70%) after each test [29].

### Sampling

At the end of the 4^th^ week, testicular samples (*n* = 5 per group) were taken for biochemical and gene expression analyses. Part of the left testis of each rat was homogenized in cold phosphate buffer saline (PBS) centrifuged at 3000× *g* for 10 min at 4 °C. The clear supernatants were kept at -20 °C for biochemical assays. Another part from the left testis was kept frozen at -80 °C for gene expression analysis.

### Biochemical assays

The serum glucose levels in control and treated groups were determined colorimetrically by a kit produced by Biodiagnostic Co., Giza, Egypt.

Testicular testosterone (ELISA; DRG Diagnostics, Marburg, Germany), estradiol (ELISA; DRG Diagnostics), acid phosphatase (ACP, EC 3.1.3.2) [30], and alkaline phosphatase (ALP, EC 3.1.3.1) [31] were assessed in testicular homogenates. Testicular malondialdehyde (MDA) levels [32], nitric oxide (NO) levels [33], reduced glutathione (GSH) levels [34], and total superoxide dismutase (T.SOD, EC 1.15.1.1) activities [35] were also determined to evaluate oxidative stress and antioxidant status in testicular homogenates. The protein levels in testicular homogenates were determined by Bradford [36] for standardization.

### RNA extraction and reverse transcription-polymerase chain reaction (RT-PCR)

Total RNA contents were extracted from testicular tissue samples in 1 mL QIAzol (79306, QIAGEN Inc., Valencia, CA, USA) with chloroform. RNA in samples was transcripted to the corresponding cDNA with RevertAid Premium reverse transcriptase (EP0733, ThermoFisher Scientific, Darmstadt, Germany). The primer sequences for cytochrome P450 17A1 (*CYP17A1*), Acetyl-CoA acetyltransferase 2 (*ACAT2*), Bcl-2-associated X (*Bax*), B-cell lymphoma 2 (*Bcl2*), Interleukin 1 beta (*IL-1β*), and glyceraldehyde 3-phosphate dehydrogenase (*GAPDH*; housekeeping) genes are listed in Table 1. To estimate the variation of gene expression on the RNA of the different samples, the CT of each sample was compared with that of the positive control group per the "ΔΔCt" method by using the following ratio: (2^-ΔΔct^) [37].

**Table 1:** Primers sequences.

Genes	5'-3' primer sequence	Accession number	References
***CYP17A1***	F: ACTGAGGGTATCGTGGATGC	NM_012753.2	[63]
	R: TCGAACTTCTCCCTGCACTT		
***ACAT2***	F: CACAACCTGGGGACAACTG	NM_001006995.1	[64]
	R: AAGGCACACGGCTTTTAGG		
***Bax***	F: GGCGAATTGGCGATGAACTG	NM_017059	[65]
	R: ATGGTTCTGATCAGCTCGGG		
***Bcl-2***	F: GATTGTGGCCTTCTTTGAGT	NM_016993	[66]
	R: ATAGTTCCACAAAGGCATCC		
***IL-1β***	F: CACCTCTCAAGCAGAGCACAG	NM_031512.2	[67]
	R: GGGTTCCATGGTGAAGTCAAC		
***GAPDH***	F: TCAAGAAGGTGGTGAAGCAG	NM_017008.4	[68]
	R: AGGTGGAAGAATGGGAGTTG		

*ACAT2*, acetyl-CoA acetyltransferase 2; *Bax*, Bcl-2-associated X; *Bcl2*, B-cell lymphoma 2; *CYP17A1*, cytochrome P450 17A1; *GAPDH*, glyceraldehyde 3-phosphate dehydrogenase; *IL-1β*, interleukin 1 beta.

### Statistical analyses

Statistical analyses of obtained data were analysed with One-way ANOVA, Tukey's post hoc multiple range tests by GraphPad Prism 5 (San Diego, CA, USA). All declarations of significance depended on *P* < 0.05.

## Results

### Open field test

**Fig 1:**
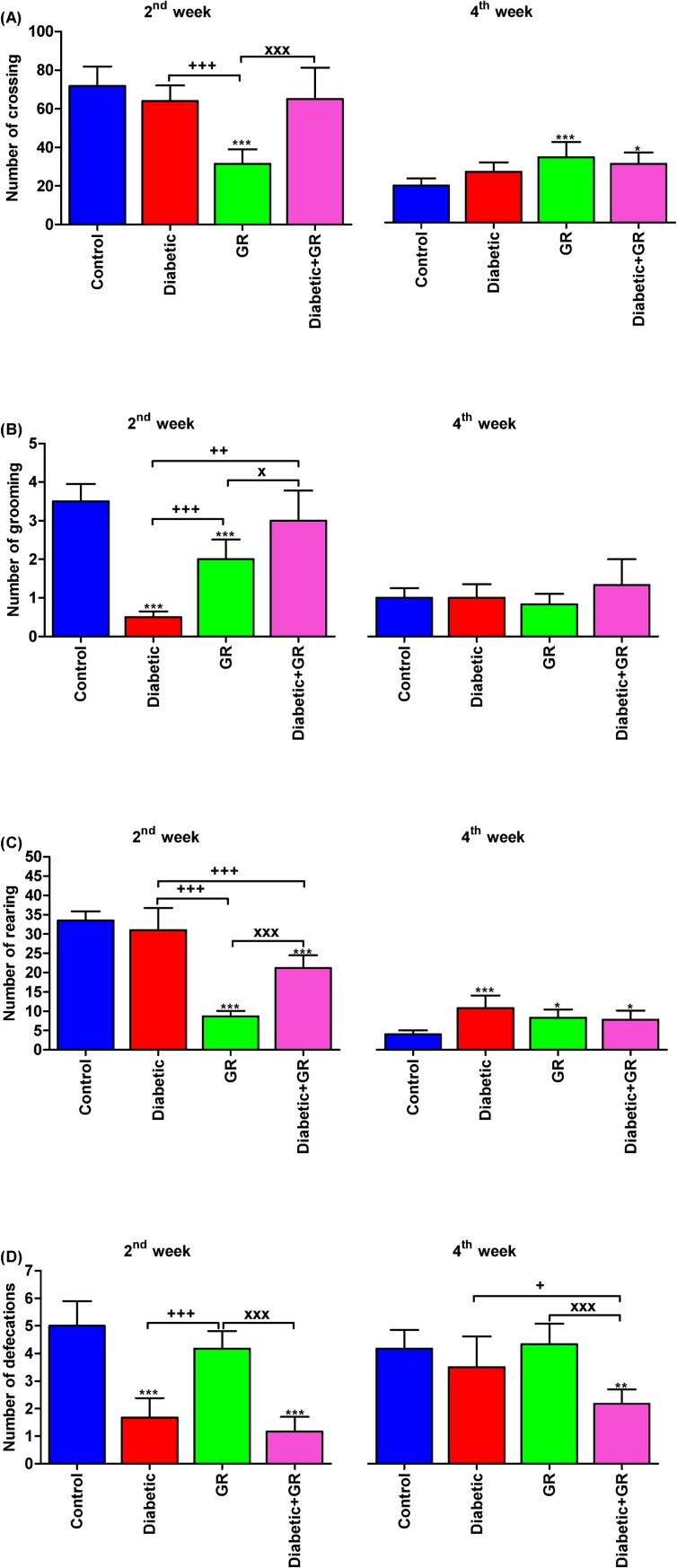
Open field test in diabetic and graviola-treated rats. **(A) number of crossing, (B) number of grooming, (C) number of rearing, and (D) number of defecations.** ^*^P < 0.*05*, ^**^P < 0.*01*, and ^***^P < 0.*001* vs. Control. ^*+*^*P < 0*.*05*, ^*++*^*P < 0*.*01*, and ^*+++*^*P < 0*.*001* vs. Diabetic. ^*x*^*P < 0*.*05* and ^*xxx*^*P < 0*.*001* vs. Diabetic+GR. Statistical analysis was performed using one-way ANOVA and Tukey's post hoc test for multiple comparisons. GR, graviola.

The OFT was used to estimate locomotor activity and exploratory behaviour (Fig 1). Results for graviola treatment on diabetic rat at the end of the 2^nd^ week in the OFT revealed that the motor activity of rats, which was represented by the number of crossing reached at its highest number in the control group compared with Diabetic or Diabetic+GR groups, showed the same level (*P* < 0.05), while the GR group showed the lowest number. Of note, exposure to OFT represents the degree of stress on the rodent. Moreover, the frequency of rearing behaviour in the GR group showed the lowest level (*P* < 0.01) in comparison with control, but treatment with graviola resulted in a slight increase in rearing but still lower than that of Diabetic group, which enhanced the pharmacological effect of graviola on stress relief.

Diabetic rats showed a significant decrease in grooming frequency as compared with control group, while treatment with graviola improved the grooming behaviour of diabetic rats toward normal. The GR group showed the same high value in grooming frequency as compared with control group.

Rats in control and GR groups had increases in the frequency of defecation in response to fear, and mild decreases were observed in Diabetic and Diabetic+GR groups. The frequency of behaviours of rats recorded in OFT test at the end of the 4^th^ week are presented in Fig 1 and indicated that the GR group had significantly impaired behavioural functions compared with age-matched controls, which appeared as an increased frequency of motor activity and defecations and rearing but lower grooming frequency. Increased anxiety levels were observed also in Diabetic and Diabetic+GR, which had a higher number of crossing and rearing than control group, but the latter had higher defecations than the other two groups.

### Elevated plus maze

**Fig 2:**
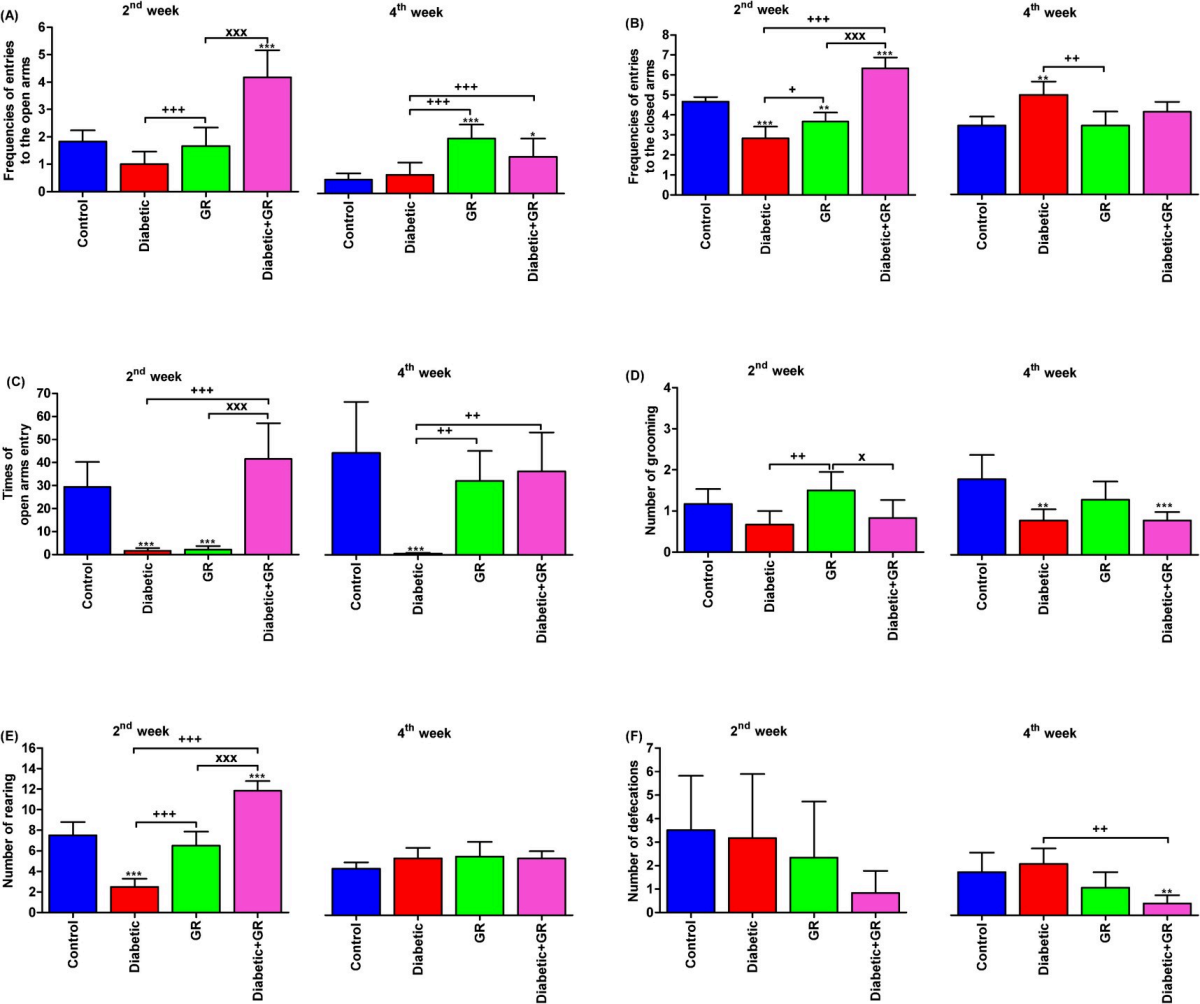
Elevated plus maze in diabetic and graviola-treated rats. **(A) frequencies of entries to the open arms, (B) frequencies of entries to the closed arms, (C) times of open arms entry, (D) number of grooming, (E) number of rearing, and (F) number of defecations.** ^*^P < 0.*05*, ^**^P < 0.*01*, and ^***^P < 0.*001* vs. Control. ^*+*^*P < 0*.*05*, ^*++*^*P < 0*.*01*, and ^*+++*^*P < 0*.*001* vs. Diabetic. ^*x*^*P < 0*.*05* and ^*xxx*^*P < 0*.*001* vs. Diabetic+GR. Statistical analysis was performed using one-way ANOVA and Tukey's post hoc test for multiple comparisons. GR, graviola.

The anxiety-like behaviours and locomotor activities were evaluated using EPM. The effects of graviola treatment on grooming, rearing, and defecation behaviours of diabetic rats at 2^nd^ week of age in EPM are given in Fig 2. The GR group showed higher grooming and lower rearing and defecations when compared with control group, although Diabetic and Diabetic+GR showed lower frequency of grooming behaviour than control group. Moreover, Diabetic+GR group tended to show the highest rearing frequency, but Diabetic group showed the lowest frequency, while GR and control groups had mild frequencies.

The results also revealed that rats in control group had slightly higher frequencies of entering the open arms and closed arms of the maze and a longer time was spent in open arms compared with GR and Diabetic rats. Diabetic+GR showed the highest frequencies of open arms and closed arms entries, and the longest time spent in open arms was also observed when compared with control group. Behavioural parameters without a normal distribution were compared between the four groups and the only significant effect (*P* < 0.05) observed was a longer time spent in open arms by the control group compared with other groups.

### Body weight and serum glucose

**Fig 3:**
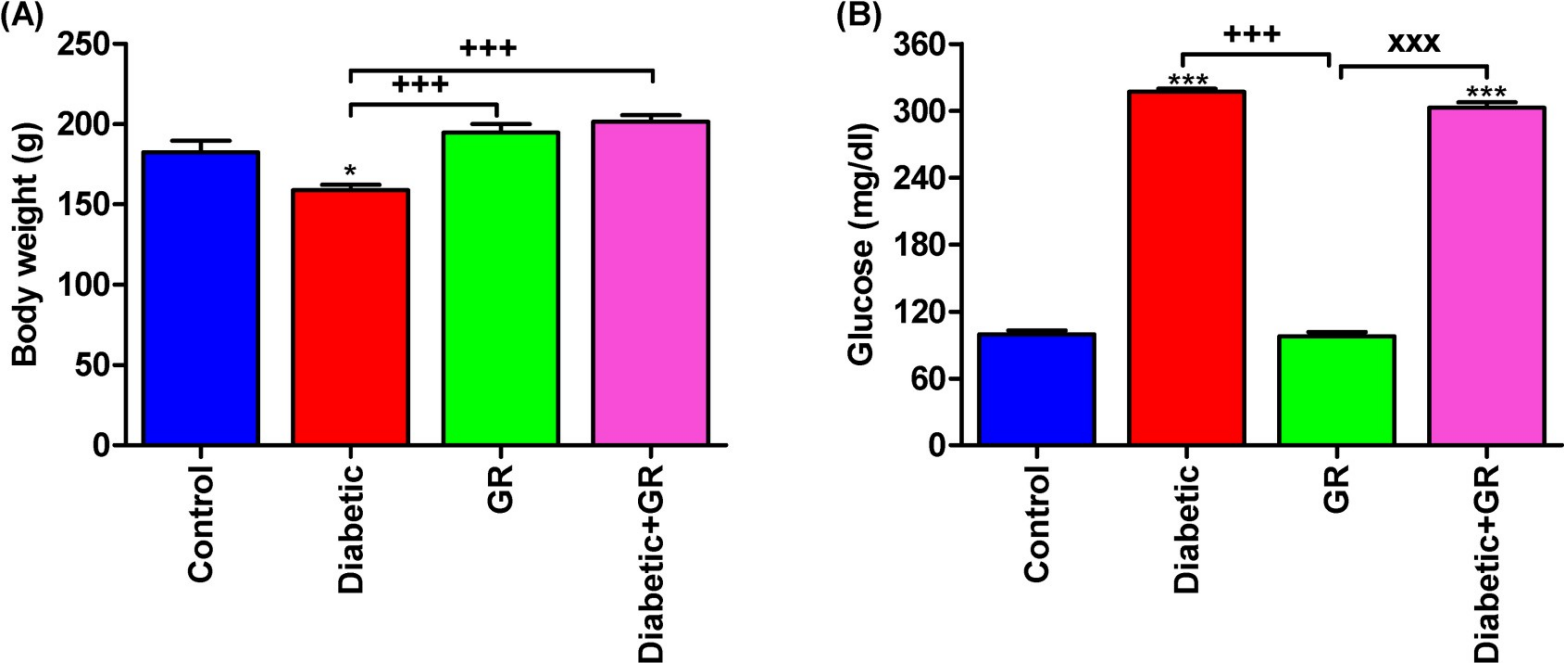
**Body weight (A) and serum glucose (B)**. The data represented that the body weights of the Diabetic group were significantly decreased compared with control, while rats in GR and Diabetic+GR groups had significant increases in their body weights in comparison with Diabetics. ^*^P < 0.*05* and ^***^P < 0.*001* vs. Control. ^*+++*^*P < 0*.*001* vs. Diabetic. ^*xxx*^*P < 0*.*001* vs. Diabetic+GR. Statistical analysis was performed using one-way ANOVA and Tukey's post hoc test for multiple comparisons. GR, graviola.

At day 30, the body weights of the Diabetic group were significantly decreased (*P* < 0.05) compared with control group (Fig 3A). Rats in GR and Diabetic+GR groups had significant increases (*P* < 0.001) in body weights in comparison with Diabetic.

Serum glucose levels were significantly increased in (*P* < 0.001) in Diabetic group, while graviola failed to decrease the elevated serum glucose level in diabetic rats supplemented with graviola (Fig 3B).

### Testicular acid phosphatase, alkaline phosphatase, testosterone, and estradiol

**Fig 4:**
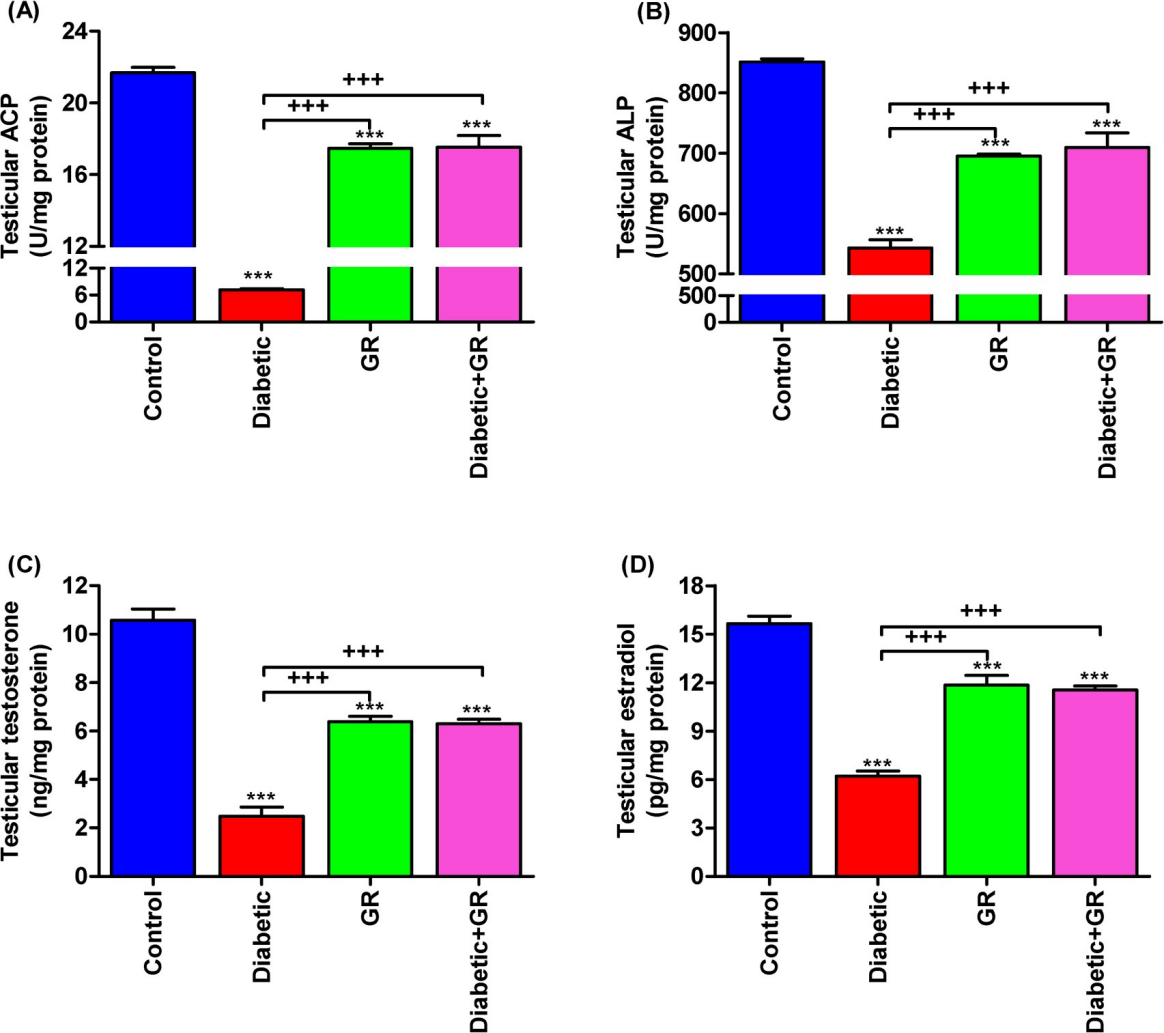
**Testicular (A) ACP, (B) ALP, (C) testosterone, and (D) estradiol levels.** Represented a restoration in testicular function that monitored by significant increases in testicular testosterone and estradiol levels in graviola-treated groups compared with diabetic rats. Samples (*n* = 5). ^***^P < 0.*001* vs. Control. ^*+++*^*P < 0*.*001* vs. Diabetic. Statistical analysis was performed using one-way ANOVA and Tukey's post hoc test for multiple comparisons. ACP, acid phosphatase. ALP, alkaline phosphatase. GR, graviola.

Results shown in Fig 4A and 4B showed significant decreases (*P* < 0.001) in testicular ACP and ALP in diabetic rats’ testicular homogenates when compared with control and graviola-treated groups. Also, the data illustrated in Fig 4C and 4D showed a significant decrease (*P* < 0.001) in testicular testosterone and estradiol levels in diabetic rats compared with other groups (*P* < 0.001). However, the graviola-treated groups showed significant increases (*P* < 0.001) in testicular testosterone and estradiol levels compared with diabetic rats.

### Testicular oxidative stress and antioxidant status

**Fig 5:**
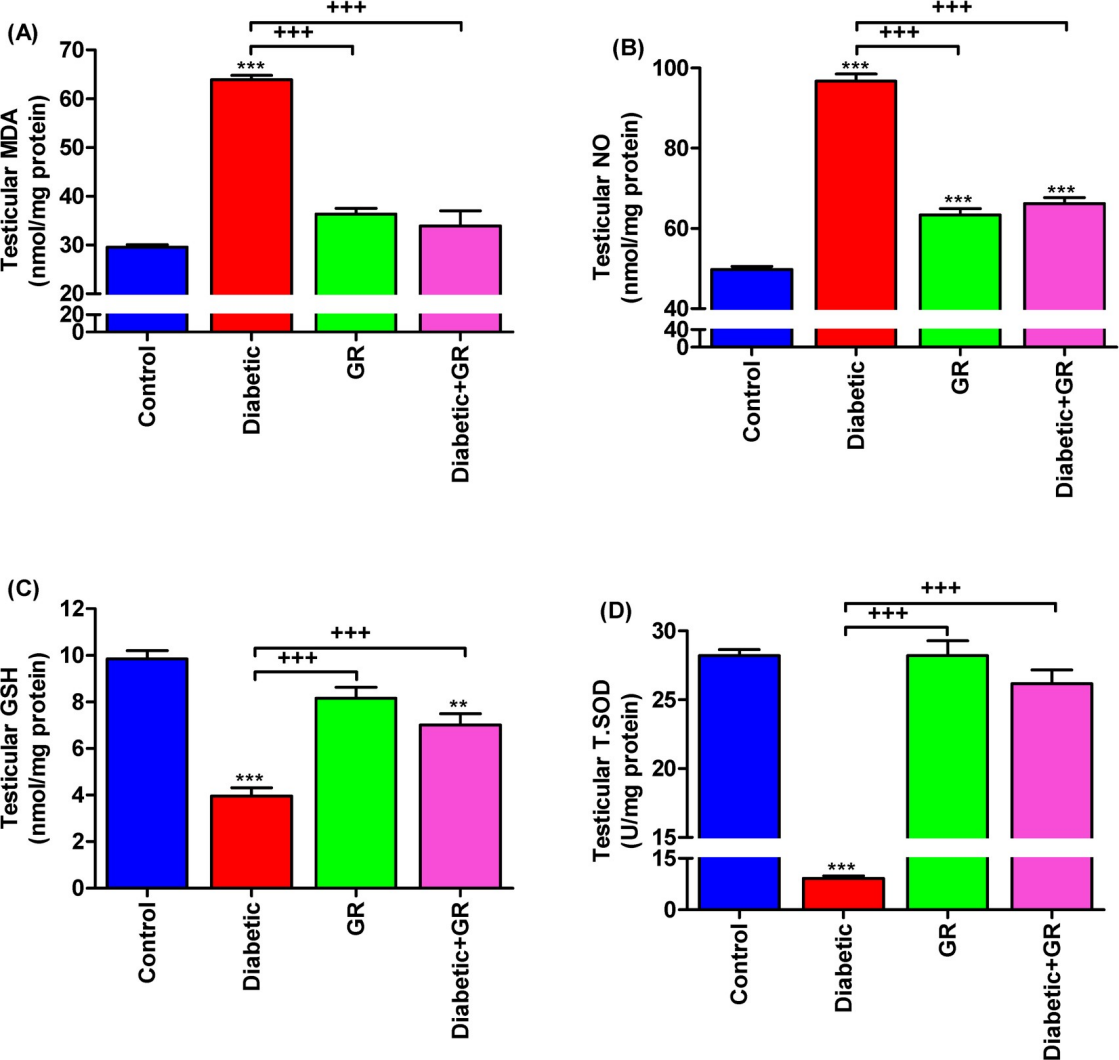
**Testicular (A) MDA, (B) NO, (C) GSH, and (D) T.SOD levels.** Graviola supplementation to diabetic rats significantly restored the testicular GSH levels and T.SOD activities that depleted due to diabetes. Samples (*n* = 5). ^**^P < 0.*01* and ^***^P < 0.*001* vs. Control. ^*+++*^*P < 0*.*001* vs. Diabetic. Statistical analysis was performed using one-way ANOVA and Tukey's post hoc test for multiple comparisons. MDA, malondialdehyde. NO, nitric oxide. GSH, reduced glutathione. T.SOD, Total superoxide dismutase. GR, graviola.

Diabetic rats showed significant increases (*P* < 0.001) in testicular MDA and NO levels compared with the control group (Fig 5A and 5B), while testicular GSH levels in Diabetic rats were significantly (*P* < 0.05) lowered (Fig 5C). Additionally, graviola administration to diabetic rats significantly restored testicular GSH (*P < 0*.*001*) levels and T.SOD (*P* < 0.001) activities that dropped due to diabetes.

### Gene expression analysis

**Fig 6:**
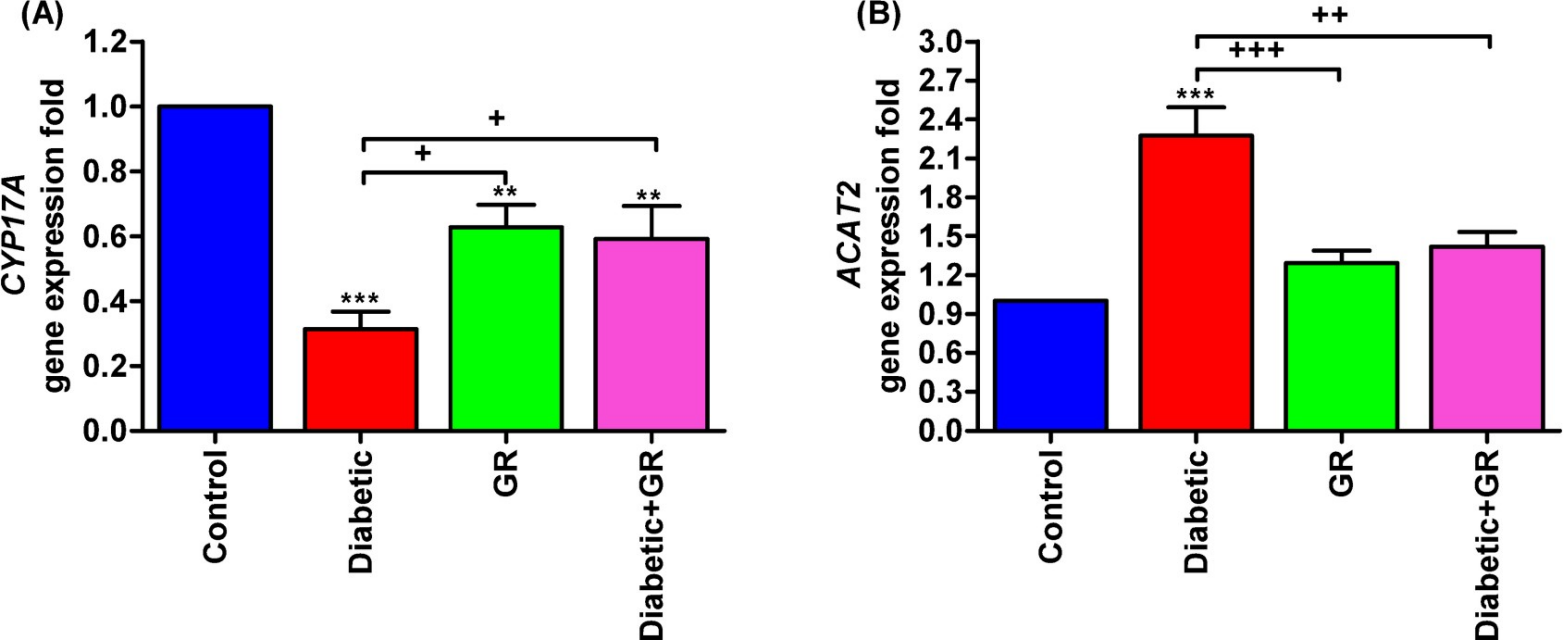
**RT-PCR validation of (A) *CYP17A1* and (B) *ACAT2*.** Graviola significantly increased the *CYP17A1* gene expression levels in graviola-treated groups that led to improvement of steroidogenesis in diabetic rats. In addition, graviola downregulated *ACAT2* that let to spare more free cholesterol for steroidogenesis. Samples (*n* = 5). ^**^P < 0.*01* and ^***^P < 0.*001* vs. Control. ^*+*^*P < 0*.*05*, ^*++*^*P < 0*.*01*, and ^*+++*^*P < 0*.*001* vs. Diabetic. Statistical analysis was performed using one-way ANOVA and Tukey's post hoc test for multiple comparisons. *CYP17A1*, cytochrome P450 17A1. *ACAT2*, acetyl-CoA acetyltransferase 2. GR, graviola.

The testicular *CYP17A1* gene expression was significantly decreased (*P* < 0.05) in the Diabetic group compared with control, as presented in Fig 6A. The graviola-treated groups showed significant increases (*P* < 0.05) in *CYP17A1* expression in comparison with Diabetic group. Testicular *ACAT2* gene was significantly expressed (*P* < 0.001) in the Diabetic rat compared with both control and graviola-treated groups. Oral supplementation of graviola to the diabetic rats caused significant decreases in *ACAT2* expressions (Fig 6B).

**Fig 7:**
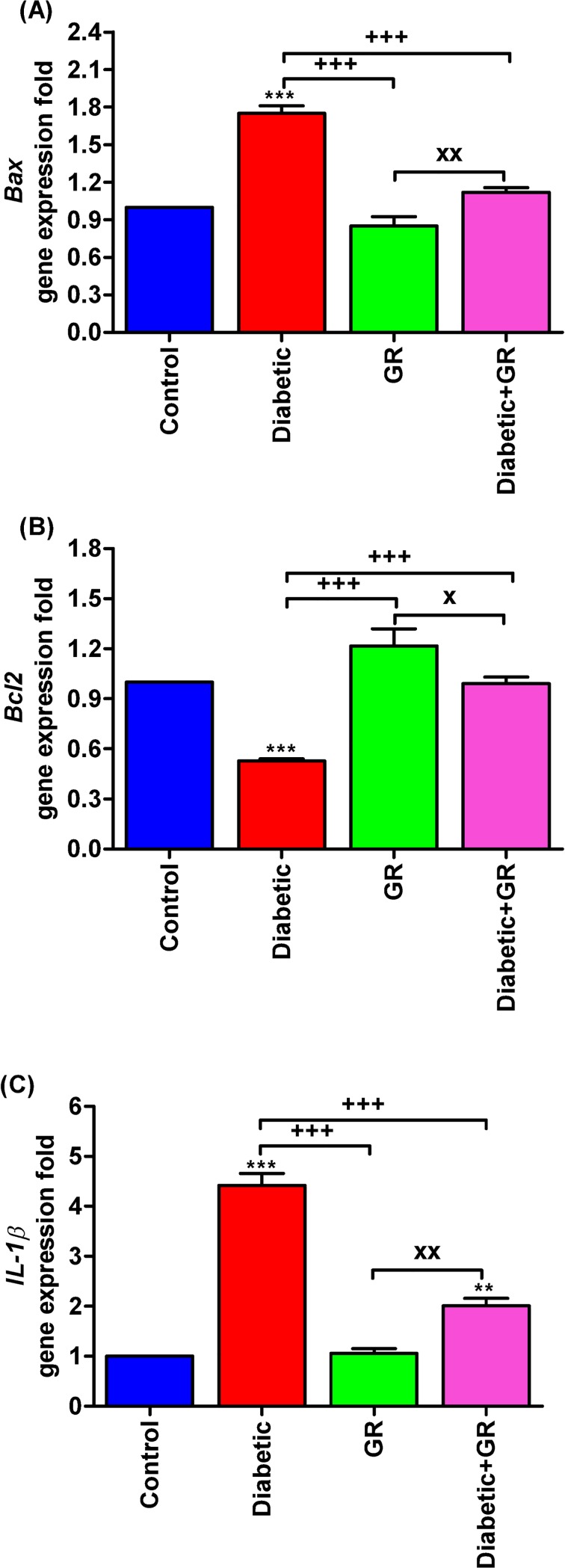
**RT-PCR validation of (A) *Bax*, (B) *Bcl2*, and (C) *IL-1β* genes.** graviola-treated rats possessed improvement in testicular tissues that evidenced by downregulations in apoptotic and inflammatory genes. Samples (*n* = 5). ^**^P < 0.*01* and ^***^P < 0.*001* vs. Control. ^*+++*^*P < 0*.*001* vs. Diabetic. ^*x*^*P < 0*.*05* and ^*xx*^*P < 0*.*01* vs. Diabetic+GR. Statistical analysis was performed using one-way ANOVA and Tukey's post hoc test for multiple comparisons. *Bax*, Bcl-2-associated X. *Bcl2*, B-cell lymphoma 2. *IL-1β*, interleukin 1 beta. GR, graviola.

Testicular *Bax* and *IL-1β* gene expressions were significantly increased (*P* < 0.001) in Diabetic rat compared with both control and graviola-treated groups. Oral supplementation of graviola to the diabetic rats caused significant decreases (*P < 0*.*05*) of *Bax* and *IL-1β* expressions (Fig 7A and 7C) than Diabetic. The testicular *Bcl2* gene expressions were significantly lowered (*P* < 0.001) in the Diabetic group in comparison with the other groups, as presented in Fig 7B. The Diabetic+GR group showed significantly increased (*P* < 0.001) *Bcl2* when compared with Diabetic.

## Discussion

DM predisposes free radical formation that induces lipid peroxidation that impairs membrane function through decline in membrane fluidity and membrane bound proteins [38,39]. DM is usually accompanied by cellular complications, which leads to morbidity and mortality in diabetic individuals [40].

Induction of diabetes in by STZ by a dose of 65 mg/kg brought testicular oxidative stress that monitored by significant increases in oxidative and inflammatory markers beside impairment of the testicular histological sections [24]. These results of our previous study encourage us to find a feed additive supplement of natural origin, like Graviola, that could counteract the oxidative and inflammatory processes in testicular tissues.

### Body weight and serum glucose

The data of the current study revealed significant decreases in rats’ body weights due to diabetes. These results came from the decline in insulin secretion in STZ-treated rats, leading to skeletal muscle catabolism along with a decline in protein synthesis and nitrogen pool balance [41].

Serum glucose levels were significantly increased in diabetic rats when compared with control group. Graviola supplementation to diabetic rats in group Diabetic+GR induced no significant changes in serum glucose levels in comparison with Diabetic group. The hypoglycemic effect of graviola may be accomplished in higher doses of graviola supplementation to diabetic rats.

### Open field tests and elevated plus maze

This study confirms that DM plays a role in the development of depression-like behaviour and anxiety in diabetic patients. Induction of diabetes with STZ altered locomotor activity in the OFT. Graviola administration attenuated the anxiogenic behaviour, as shown by the increase of total distance crossed and the decrease of the immobility time. In addition, diabetic rats showed a high immobility time compared with non-diabetics, reflecting despair-like behaviour [42]. In the current study, we have noted that graviola administration in diabetic rats improved locomotor activity in the OFT, according to the increased total distance crossed and the decreased immobility time. Our study demonstrated clearly the antidepressant-like effect of graviola in diabetic rats. The role of graviola in the modulation of several neurotransmitter systems, such as serotonin, GABA and nitric oxide has also been studied [43].

Moreover, the results of OFT revealed great anxiolytic effect of graviola treatment on control and diabetic rats during the 2^nd^ and 4^th^ weeks of age. By reviewing the OFT records, we noted a high number of crossings, thus we conclude that there was not any motor deficit. In addition, Carter and Shieh [44] illustrated that during senility phase, motor reflexes decrease.

Also, we documented the following (a) at the end of the 2^nd^ week of age no significant difference between crossing numbers of diabetic rats versus graviola treated group; (b) at 4^th^ week of age we observed that there was mild increase in crossing numbers in GR and Diabetic+GR groups compared with the rest of the groups. This might be attributed to the rise in anxiety level. This correlated with Aucoin and Bhardwaj [45] observations that anxiety and impulsivity were worsened due to cerebral hypoglycemia.

Higher grooming frequency resulted in better appearance of graviola treated group than diabetic group, however, we note that grooming was often interpreted as displacement behavior and self-calming procedure [46]. Also, during the 2^nd^ and 4^th^ weeks we noted lower rearing. Also, an increase in fear response has been associated with an increase in frequency of defecation in the OFT test. According to our results, Diabetic+GR group had lower defecation frequency, which decreased the stress level. Sautou-Miranda *et al* [47] attributed this to the stability of dopamine.

On one hand, the EPM results of the 2^nd^ week revealed increased anxiety in control and Diabetic rats compared with Diabetic+GR rats, and this was evident through decreased numbers of OA, EA entries and less time spent in the OA, suggesting motor activity changes in diabetic rats. The ethological measure such as grooming behavior showed nonsignificant differences between groups, although Diabetic+GR group had the highest rearing frequency, which might be attributed to the height of the maze that gave rats the opportunity for vertical movements (climbing, hanging, and jumping) due to their anxiolytic state to explore the maze. Also, a lower number of excreted boli ensured decreased anxiety in Diabetic+GR or GR rats. On the other hand, data analysis of the EPM test during the 4^th^ week showed nonsignificant differences between groups in all behaviors except time in OA, in which control rats had the highest level; this might attributed to past experience, which led to change in effectiveness of anxiolytic drugs similar to Cryan and Sweeney [48] who noted changed anxiety state between two trials as a result of triggering the behavior from unconditioned fear avoidance to learned avoidance based on prior exposure to the situation.

### Testicular acid phosphatase, alkaline phosphatase, testosterone, and estradiol

ALP and ACP are intracellular enzymes present in the testicular tissues. Therefore, the decline in their levels indicates testicular cellular damages mainly due to oxidative injuries such as those accompanying DM that led to severe reproductive alterations [49]. Spermatogenesis and testicular physiology have been promoted by testicular testosterone, while estradiol is responsible for protection of male sperm cells from apoptosis [50,51]. In our investigation, diabetic rats had significant decreases in both testicular testosterone and estradiol. The same results were stated by Farrell *et al* [52]. Oxidative damages in Leydig cells led to severe decline in testicular testosterone in diabetic rats [53]. On the contrary, graviola perfectly protects the testicular tissues from oxidative deteriorations and maintained the testicular testosterone and estradiol levels.

This antioxidant potential of graviola may be regarded to phenols, flavonoids, vitamins, and carotenoids [18]. Catechin, kaempferol, quercetin, and caffeic acid are the most flavonoid that present in graviola and other medicinal plants of antioxidant activities in diabetes [54–56]. Also, graviola contains vitamins c and E that can perfectly scavenge the generated free radicals in diabetics [57].

### Testicular oxidative stress and antioxidant status

The data of four weeks of graviola supplementation to STZ-induced diabetic rats revealed significant improvement of rat behaviour and testicular antioxidant status. The antioxidant potential of graviola is considered as the crucial mechanism for protection of rats from diabetic alterations in rat’s behaviour and testicular reproductivity. This data mainly focused on the reproductive benefits of graviola treatment in the setting of diabetes, with the conclusion that graviola is protective against free radicals in this testis. Previous studies stated the antioxidant potential of graviola diabetic rat’s liver through enhancement of GSH level and SOD activities [58]. Also, Bauche *et al* [59] reported a remarkable rise in MDA levels in rats of induced diabetes. This result indicated the protective effect of graviola against the harmful effects of DM through significant enhancement in testicular antioxidant enzymes.

### Gene expression analysis

The *CYP17A1* gene encodes the cytochrome P450c17 enzyme that is a regulator of steroidogenesis in adrenal gland, gonads, and placenta of rodents [60]. Results of the current study revealed a significant decrease in *CYP17A1* gene in Diabetic group that led to impairment in testicular testosterone, while graviola significantly increased this gene expression levels in graviola-treated groups compared with Diabetic one.

Testicular *ACAT2* gene is a part of cholesterol homeostasis in testis that is stimulated by delivery of cholesterol to the endoplasmic reticulum that leads to cholesterol esterification [61]. Overexpression of *ACAT2* leads to dysregulation of testicular cholesterol metabolism. Rats in the graviola-treated group successfully decreased the levels of *ACAT2* gene expression near to the control group level.

Oxidative injuries accompanying DM lead to expression of *IL-1β*, the inflammatory mediator [24] and to the apoptotic gene *Bax*. The overexpression of both genes led to induction of pro-apoptosis in diabetic rats. Moreover, the diabetic rats possessed lower expression of the anti-apoptotic gene *Bcl2* [62]. Concomitantly, the apoptotic process increased in testicular tissues of diabetic rats, while graviola-treated rats were protected against diabetic apoptosis and inflammatory process.

## Conclusion

Oxidative stress induces alterations in diabetic rats’ behaviour and testicular injuries. Graviola supplementations overcame these alterations through modulation of diabetic rats’ behaviour along with enhancement of testicular antioxidant status that was accompanied by reduction in inflammatory markers and cellular apoptosis. In addition, graviola improved the hormonal reproductive potential of diabetic rats. To qualify this effect that was found in diabetic male rats, clinical studies in human are required to validate animal studies

## Supporting information

S1 File Raw data. (PZF)

## Abbreviations

ACAT2: Acetyl-CoA acetyltransferase 2
ACP: Acid phosphatase
ALP: Alkaline phosphatase
Bax: B-cell lymphoma 2
Bcl2: Bcl-2-associated X
CYP17A1: Cytochrome P450 17A1
EPM: Elevated plus maze
GAPDH: Glyceraldehyde 3-phosphate dehydrogenase
GSH: Reduced glutathione
IL-1β: Interleukin 1 beta
MDA: Malondialdehyde
NO: Nitric oxide
OFT: Open field test
ROS: Reactive oxygen species
STZ: Streptozotocin
T.SOD: Total superoxide dismutase
